# Phenological response of European beech (*Fagus sylvatica* L.) to climate change in the Western Carpathian climatic-geographical zones

**DOI:** 10.3389/fpls.2024.1242695

**Published:** 2024-04-03

**Authors:** Jana Skvareninova, Roman Sitko, Jaroslav Vido, Zora Snopková, Jaroslav Skvarenina

**Affiliations:** ^1^ Faculty of Ecology and Environmental Sciences, Technical University in Zvolen, Zvolen, Slovakia; ^2^ Faculty of Forestry, Technical University in Zvolen, Zvolen, Slovakia; ^3^ Slovak Hydrometeorological Institut, Banská Bystrica, Slovakia

**Keywords:** phenology, European beech, climate change, Western Carpathians, trend, relief, elevation, heterogeneity

## Abstract

**Introduction:**

The paper analyzes the results of 26 years (1996–2021) of phenological observations of the vegetative organs of European beech (*Fagus sylvatica* L.) in the Western Carpathians. It evaluates the influence of the heterogeneity of this territory, including relief and elevation, based on climatic-geographical types.

**Methods:**

Phenological stages, including leaf unfolding, full leaves, leaf coloring, and leaf fall, were monitored at 40 phenological stations across eight elevation zones. The study assesses trends in the occurrence of phenological stages, the length of the growing season, and phenological elevation gradients.

**Results:**

The results indicate a statistically significant earlier onset of spring phenological phases and delay in autumn phases, resulting in an average extension of the beech growing season by 12 days. Our findings confirm that the lengthening of the growing season due to warming, as an expression of climate change, is predominantly attributed to the warming in the spring months. The detected delayed onset of autumn phenophases was not due to warming in the autumn months, but other environmental factors influence it. The trend of elongation of the growing season (p<0.01) is observed in all elevation zones, with a less significant trend observed only in zones around 400 and 600 m a.s.l, signaling changes in environmental conditions across most of the elevation spectrum. Moreover, the heterogeneity of climatic-geographical types within each elevation zone increases the variability in the duration of the growing season for sites with similar elevations. By extending the growing season, it is assumed that the beech area will be changed to locations with optimal environmental conditions, especially in terms of adverse climatic events (late spring frosts, drought) during the growing season. The phenological elevation gradients reveal an earlier onset of 2.2 days per 100 m for spring phenophases and a delay of 1.1–2.9 days per 100 m for autumn phenophases.

**Discussion:**

These findings highlight the specific environmental conditions of European beech in the Western Carpathians and their potential for anticipating changes in its original area. Additionally, these observations can aid in forecasting the further development of phenological manifestations related to climate change.

## 1 Introduction

European beech (*Fagus sylvatica* L.) growths in regions with favorable growth conditions characterized by oceanic and transitional oceanic-continental climates. This suboceanic species exhibits a wide distribution across Europe, predominantly in natural forest communities in cooler climates. Beech naturally avoids frost-prone areas with frequent temperature inversions (cold-air tailback) (Dittmar and Elling, 2006) and is sensitive to late spring frosts (Menzel et al., 2015). Beech trees have specific thermal requirements and, therefore, favor well-balanced temperature conditions, which are within the Carpathian massif, more commonly found in mid-mountain regions with a more oceanic climate. Conversely, low temperatures in certain higher mountainous areas limit the presence of beech in forest stands, and it is not typically found growing in the hottest and driest lowland regions because European beech is relatively sensitive to drought (Backes and Leuschner, 2000; Lukasova et al., 2014; Vido et al., 2016, Lukasová et al., 2021). In the mountainous regions of the Western Carpathians, beech trees thrive with an average annual temperature of 10°C and annual precipitation ranging from 800 to 1000 mm (Bialobok et al., 1990). In addition to temperature and precipitation, the distribution of European beech also depends on other geographical factors, including exposure and the ruggedness of the terrain. Vegetation zonality aligns with the climate zones, and in regions with varying elevations occurs vertical gradations in vegetation. Vegetation elevation gradation represents the vertical stratification of ecosystems in mountainous areas. Climate changes lead to natural (climax) variations in vegetation with increasing elevation and in response to specific exposures and slope positions (Arnič et al., 2021). Geographical elevation gradation corresponds to changes in the local climate and significantly influences vegetation gradation (Zlatník, 1974 referenced in Škvarenina et al., 2004).

Numerous studies dating back to the early 21st century (Chmielewski and Rötzer, 2001; Walther et al., 2002; Menzel et al., 2006; Bertin, 2008; Malyshev et al., 2022) have evaluated the impact of gradual climate change by observing dynamic phenological changes in plants and trees. For example, meteorological droughts have been linked to an earlier onset of leaf senescence and a shortened growing season (Chuchma et al., 2016; Středová et al., 2021), underscoring the importance of phenology as a bioindicator in today’s changing ecosystems (Rafferty et al., 2020; Wenden et al., 2020. Phenology is crucial for assessing the impact of climate change on terrestrial ecosystems’ development. Trends of earlier spring leaf development and delayed autumn leaf coloring, as manifestations of this change, are likely to influence the competitive balance among tree species. They enable the prediction of seasonal shifts in the 21st century in response to climate warming (Vitasse et al., 2011).

Long-term phenological records of tree species provide valuable insights into their biological responses to climate change in temperate regions with distinct seasons. These records are crucial for identifying trends in phenological phase development and their relationship to climate change (Asse et al., 2018; Škvareninová and Mrekaj, 2022). Dependency of phenological variability in long-term time series of several plant species suggest significant changes in the growing season and the spatial distribution of species (Kariyeva et al., 2012).

Phenological phases, such as leaf unfolding and leaf coloring, serve as important indicators that can be utilized to assess the local adaptation of trees to regional climatic conditions and to monitor climate-related changes in nature. Annual phenological changes represent a strategy for adapting to weather fluctuations and coping with a wide range of environmental conditions in a changing climate (Capdevielle-Vargas et al., 2015).

Several phenological models (Melaas et al., 2013; Meng et al., 2021) incorporate temperature and photoperiod as limiting factors influencing spring and autumn phenophases. Sitko et al. (2022) compared such models and found that photoperiod is a critical factor for improving predictions of spring phenology. Delpierre et al. (2009) also noted that photoperiod is a triggering driver of senescence, with its progression being influenced by cold temperatures.

Leaf phenology is one of the most reliable bioindicators of ongoing global warming in temperate and boreal zones due to its high sensitivity to temperature fluctuations (Fu et al., 2019). Temperature plays a pivotal role in triggering tree activity in spring. For instance, Basler and Körner (2012) identified photoperiod as a constant and dependable indicator of the onset of the growing season, regardless of the weather fluctuations. They observed that early-succession species exhibited no significant photoperiod sensitivity, while late-successional tree species relied heavily on photoperiod as an environmental cue. As temperatures rise, phenology will shift towards species-specific photoperiod thresholds.

There is no clear dominant environmental driver in the cessation of tree activity in autumn and the onset of dormancy. However, temperature, photoperiod, and water stress emerge as prominent factors, as noted by Delpierre et al. (2016). Beyond air temperature, the autumn phenological phases of beech trees are significantly influenced by precipitation, particularly during summer. Water deficits, leading to early leaf coloring, can shorten the growing season, potentially altering competitive relationships between tree species and entire ecosystems in the years ahead (Bolliger et al., 2000; Gessler et al., 2007; Meier et al., 2011; Jantsch et al., 2013). The sensitivity of beech trees to meteorological changes is also underscored by Augustaitis et al. (2012). Increasingly, research focuses on the phenological manifestations of beech trees influenced by elevation (Vitasse et al., 2009; Schieber et al., 2013; Popescu and Sofletea, 2020). The extent to which meteorological elements change with increasing elevation can be elucidated through the phenological elevation gradient (Dittmar and Elling, 2006). Consequently, the phenological gradient is a suitable bioindicator of climate change when comparing various but equally long periods (Čufar et al., 2012; Škvareninová, 2013, 2016). Phenological observations of beech are crucial, particularly in areas where frost resistance is a determining factor. Středa et al. (2011) analyzed stand microclimates, emphasizing the role of microclimatic monitoring, especially in research related to late frosts.

This study aims to analyze the long-term (1996-2021) phenological patterns of common beech in response to climate change, employing phenological bioindicators across various environmental conditions in the Western Carpathians. The primary bioindicators selected for this analysis include trends in the onset of vegetative phenophases, the phenological elevation gradient, and the duration of the growing season. Given the substantial heterogeneity of the territory and its impact on beech phenological phases, a climatic-geographical classification was employed. This classification was used to explain certain phenological anomalies observed at different elevations. We addressed the following study questions: i) What are the trends in the timing of spring and autumn phenological phases of European beech in the Western Carpathians over the 26-year period from 1996 to 2021? ii) Has the length of the growing season for European beech in the Western Carpathians increased, and how significant are these changes across different elevation zones? iii) How does the heterogeneity of climatic-geographical types within each elevation zone affect the variability in the duration of the growing season for sites with similar elevations? iv) What is the relationship between elevation and the onset of spring and autumn phenophases for European beech in the Western Carpathians? v) Does warming in the spring and autumn months affect the lengthening of the growing season across all elevation zones?

## 2 Materials and methods

### 2.1 Study area

**Figure 1:**
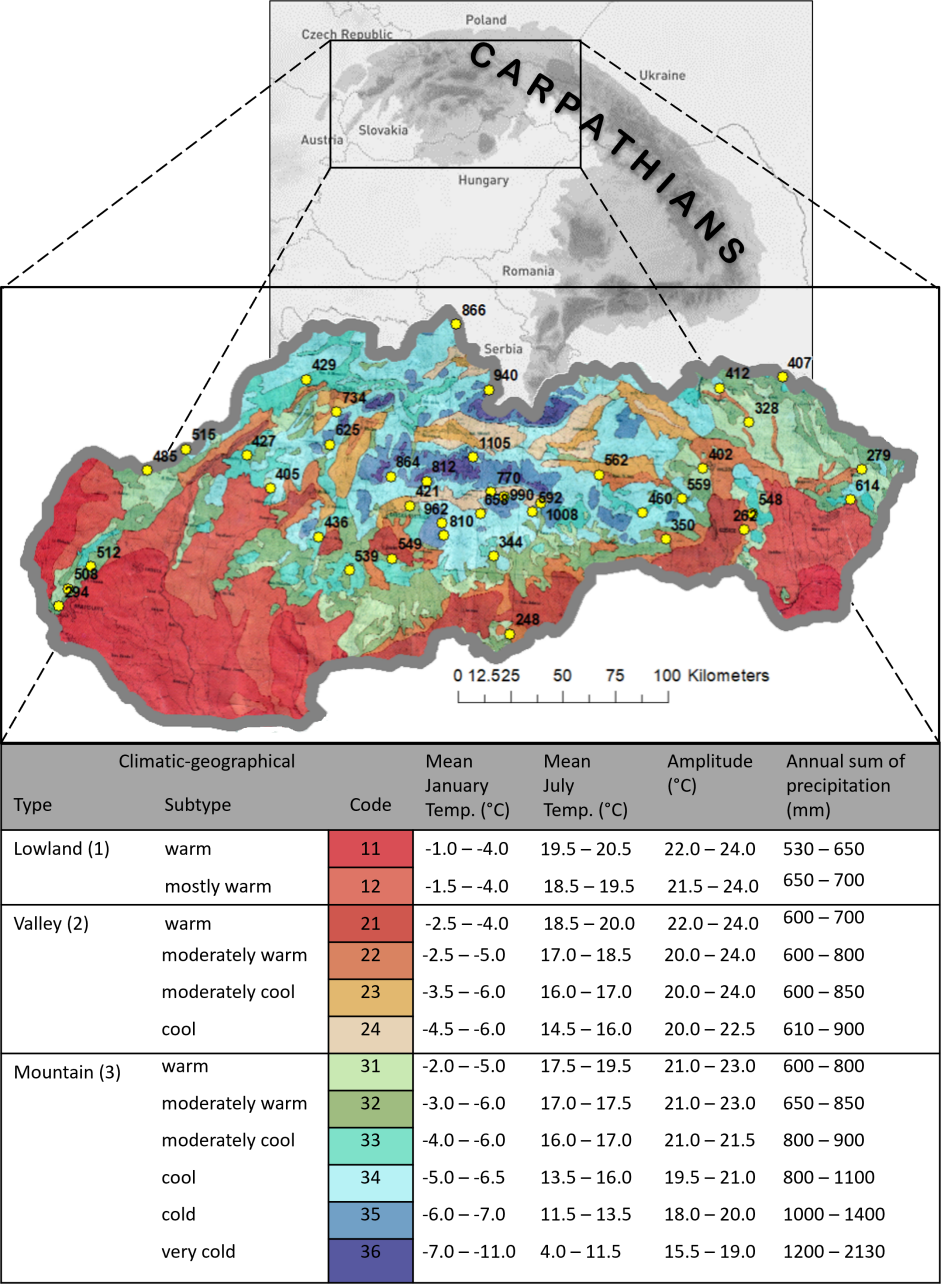
Climatic-geographical types and subtypes of Slovakia (Tarábek, 1982) with position (yellow dots) and elevation of used phenological observational sites.

The study area is in Central Europe and covers most of the Western Carpathians (**Figure 1**). According to biogeography, the area falls within the transitional Atlantic-continental Central European climate (Alisov and Poktarus, 1974). Due to the substantial presence of the Carpathian range, the climate in this area is primarily shaped by orography. The differentiation of elevation across the territory and the influence of the relief represent significant factors in climate formation. The conventional Köppen classification (Beck et al., 2018) and the classification by Alisov and Poktarus (1974) divide the Western Carpathians relatively coarse. Therefore, the regional climate-geographical classification by Tarábek (1982) was utilized for purposes of phenological analyses. This classification is widely used in geographic, biological, and ecological studies of the region (e.g., Bublinec and Pichler, 2001; Hrivnák and Ujhazy, 2005; Mihál and Blanár, 2014; Kamenišťák et al., 2020). The classification is based on fundamental climate types (lowland, valley, mountain) determined by the relief’s configuration. Within climate types, 12 subtypes were assigned based on the temperature sum of the period with average daily temperatures above 10°C and average January and July temperatures, as well as on the annual amplitude of average monthly air temperatures in the territory of Slovakia. The rationale for this approach to zonation is that in the climate of Western Carpathians, which is sufficiently humid to very humid, temperatures are the determining limiting factor in landscape processes, whereas the quantitative differentiation of moisture, which is expressed in terms of average annual precipitation, determines only the intensity of landscape processes (Tarábek, 1982).

Climatic-geographical types (**Figure 1**) represent another factor characterizing the specificity of the mountainous climate in a given area and have an impact on the phenological activity of beech. The map on a scale of 1:1,000,000 was utilized to assign climatic-geographical subtypes to every of 40 sites. Such environmental conditions are often found in the rugged terrain of the Carpathians. Therefore, we have included them in our phenological assessment. The climate influenced by the terrain relief creates significantly distinct areas with unique climatic regions. Tarábek classification (Tarábek, 1982) has become a suitable methodological approach for describing such environmental conditions. It includes locations on mountain ridges (crests) where the influence of wind and frost is evident, slopes with varying exposures and shading, and valleys where temperature inversion is the primary factor (Plesník, 2004).

Most of the Western Carpathians’ territory falls within the zone of temperate deciduous forests, with mountain locations included in the European mountain temperate forests zone due to the unique montane climate (Walter, 1977). Beech forest communities in the middle and upper mountain regions of the Carpathians create extensive ecosystems comprising mixed deciduous forests and monocultures. Many of these areas have been preserved in the form of primeval forests and have earned recognition as UNESCO World Natural Heritage sites (Kadlečík, 2016). The Western Carpathians primarily cover mountainous regions, with the lower limit of beech occurrence beginning at an elevation of 150 m in areas prone to temperature inversions with northern exposure, often coexisting with sessile oak (Hrvoľ et al., 2009). European beech thrives at elevations of 600–800 m, primarily on moist soils at the base of slopes (National Forest Centre, 2015; Lukasová et al., 2019), forming large, homogeneous stands. At higher elevations, it shares its habitat with silver fir and Norway spruce, with species representation varying according to environmental conditions. The average upper limit for its occurrence reaches 1100 m.

Within the study area, 40 sites with phenological observations were selected for analysis. During site selection (**Figure 1**), vertical zonation was considered to include a diverse range of sites within different elevation zones that encompass the entire area of mass occurrence of beech. Sites with short time series of observations were excluded during data processing from analysis. From the total number of observations at 40 stations over 26 years (1040 observations), 98-99% of observations were available for analysis within individual phenophases. The number of observation sites and their distribution within individual elevation zones are described in **Table 1**.

**Table 1:** Distribution of phenological observations sites and protected areas of beech within the elevation zones.

Name of protected area (PA)	Number of PA	Elevation zone	Elevation range of observation sites (m a.s.l.)	Number of observation sites
*Cerová vrchovina, *Malé Karpaty	2	300	248–344	5
*Východné Karpaty, **Slovenský kras	2	400	350–436	9
*Biele Karpaty, *Malé Karpaty, *Štiavnické vrchy	5	500	460–549	9
*Vihorlat, **Muránska planina, **Slovenský raj	3	600	559–625	5
	0	700	658–734	2
*Poľana, **Nízke Tatry	3	800	770–812	3
*Horná Orava, **Veľká Fatra, **Vysoké Tatry	3	900	864–940	3
*Poľana, **Muránska planina, **Nízke Tatry	4	1000	962–1105	4

*Protected landscape area; **National park.

### 2.2 Leaf phenology and meteorological data

Long-term phenological observations of beech were conducted between 1996 and 2021 at elevations ranging from 248 to 1105 meters above sea level. The observations occurred within the station network established by the Slovak Hydrometeorological Institute. The stations represent the natural conditions in the Inner Western Carpathians and the part of the Outer Western Carpathians, which extends over the territory of Slovakia. The methodology for phenological observations followed the guidelines of the Slovak Hydrometeorological Institute (Braslavská and Kamenský, 1996), which aligns with the numerical codes of the international BBCH scale (Meier, 1997). This scale provides standardized phenological development stages across the European network of phenological stations. The onset of phenophases was recorded at 40 sites, each with a minimum of 10 mature trees (over 50 years old). The range of longitude within all sites is 5° (17.03°-22.22°) and in latitude slightly exceeds 1° (48.17°- 49.52°). The collected data were subsequently transformed into Julian calendar values, replacing each date of phenophase onset with the corresponding serial number representing the day of the year (DOY).

The observations of spring phenophases were ongoing from the beginning of April in lower locations to the half of May in mountainous areas. The assessment interval took place at individual sites every 5-7 days. Since the course of the autumn phases is slower than the spring phases, the observation interval is extended to 7-10 days, depending on the weather. Observations took place from the beginning of September to the end of October. The assessment focused on the following vegetative phenological phases:

- Leaf unfolding (BBCH 11 - LU): reached when the first undeveloped leaves of light green color appear on at least half of the observed group (**Supplementary Figure 1A**).
- Full leaves (BBCH 13 - FL): occurs when the first fully developed leaves appear on at least half of the observed group (**Supplementary Figure 1B**).
- Leaf coloring (BBCH 92 - LC): marked by the appearance of discolored leaves on at least half of the observed samples (**Supplementary Figure 1C**).
- Leaf fall (BBCH 93 - LF): reached when the first discolored leaves spontaneously fall to the ground.

The observations were carried out according to the methodological manual, where the phenophases were precisely defined. To eliminate the subjectivity of the observer and to correctly determine the phenophase, atlases with photographs of phenological phases were available. The initial records of the phenophases onsets underwent a professional review by a phenological expert of the Slovak Hydrometeorological Institute.

In addition to individual phenophases, the length of the growing season was also evaluated. The growing season begins with the onset of leaf unfolding and concludes with the onset of leaf coloring, indicating the cessation of physiological activity in trees. The elevational range was divided into eight zones to explore the phenological responses of beech across different elevations within the observed territory. The first (300) and last (1000) elevation zone had one assigned station that surpassed the 100 m interval used for the remaining zones (400, 500, 600, 700, 800, 900). Some of the stations are situated within protected areas (**Table 1**).

The climatic data employed in the analyses were sourced from publicly available climate databases. Monthly mean temperatures for three meteorological stations in Slovakia were acquired using the web application KNMI Climate Explorer (Climate Explorer: Starting point (knmi.nl)), and the Meteomant.com website was utilized for one station. All four meteorological stations encompass an altitude gradient ranging from 231 to 906 m above sea level. Data pertaining to average monthly temperatures for 40 sites with phenological observations were extracted from the E-OBS gridded database (Cornes et al., 2018), featuring a resolution of 0.25° of longitude and latitude. The use of this climate database type is substantiated by a previous study (Sitko et al., 2016).

### 2.3 Statistical analyses

To analyze the onsets of phenophases and the duration of the growing season, and a comparison was made between elevation zones. Phenological elevation gradients were calculated using regression analysis methods, expressing the dependence of phenophase onset or growing season duration on elevation, measured in days per 100 meters of elevation. A coefficient of determination was employed to assess the proportion of variance explained by the relation between phenological phase onset, growing season duration, and elevation. Fisher’s transformation of z statistic was used to test the significance of the Spearman correlation coefficient, and the non-parametric Mann-Kendall test was used to test the monotonical trends in the shift of phenological phase onset and growing season duration over the 26-year observation period. ANOVA with LSD *post-hoc* test was used to compare growing season duration within climatic-geographical types. The outliers identified by the Grubbs test were removed from the growing season duration dataset.

To confirm the influence of recent climate change on temperature patterns in Western Carpathian areas where European beech is present, two-month average temperatures preceding and during the onset of spring and autumn phenophases were computed from data collected at four meteorological stations covering the period 1996-2021. March and April were selected for spring phenophases, while September and October were chosen for autumn phenophases. Subsequently, means were calculated from these 2-month averages from all four stations, representing the entire elevation gradient with the occurrence of European beech in the Western Carpathians. Monotonic trends in the development of 2-month temperature mean for the period 1996-2021 were assessed using the Mann-Kendall test. Correlation analysis was employed to examine the influence of temperature on the day of the year (DOY) of the onset of spring and autumn phenophases, determining the duration of the growing season (LU and LC). From the monthly temperature means, 2-month means were recalculated, using March and April for spring phenophases and September and October for autumn phenophases. The analysis was conducted separately for individual elevation zones. Therefore, for each year from 1996-2021, the average DOY of the phenophase onset was computed from all sites in respective elevation zones, and these data were correlated with the mean of the 2-month temperature mean corresponding to these locations. The significance of the Spearman correlation coefficient was tested at p< 0.05. All analytical methods were implemented using Statistica 12 software (StatSoft, Inc, 2013).

## 3 Results

### 3.1 Spring phenology

**Figure 2:**
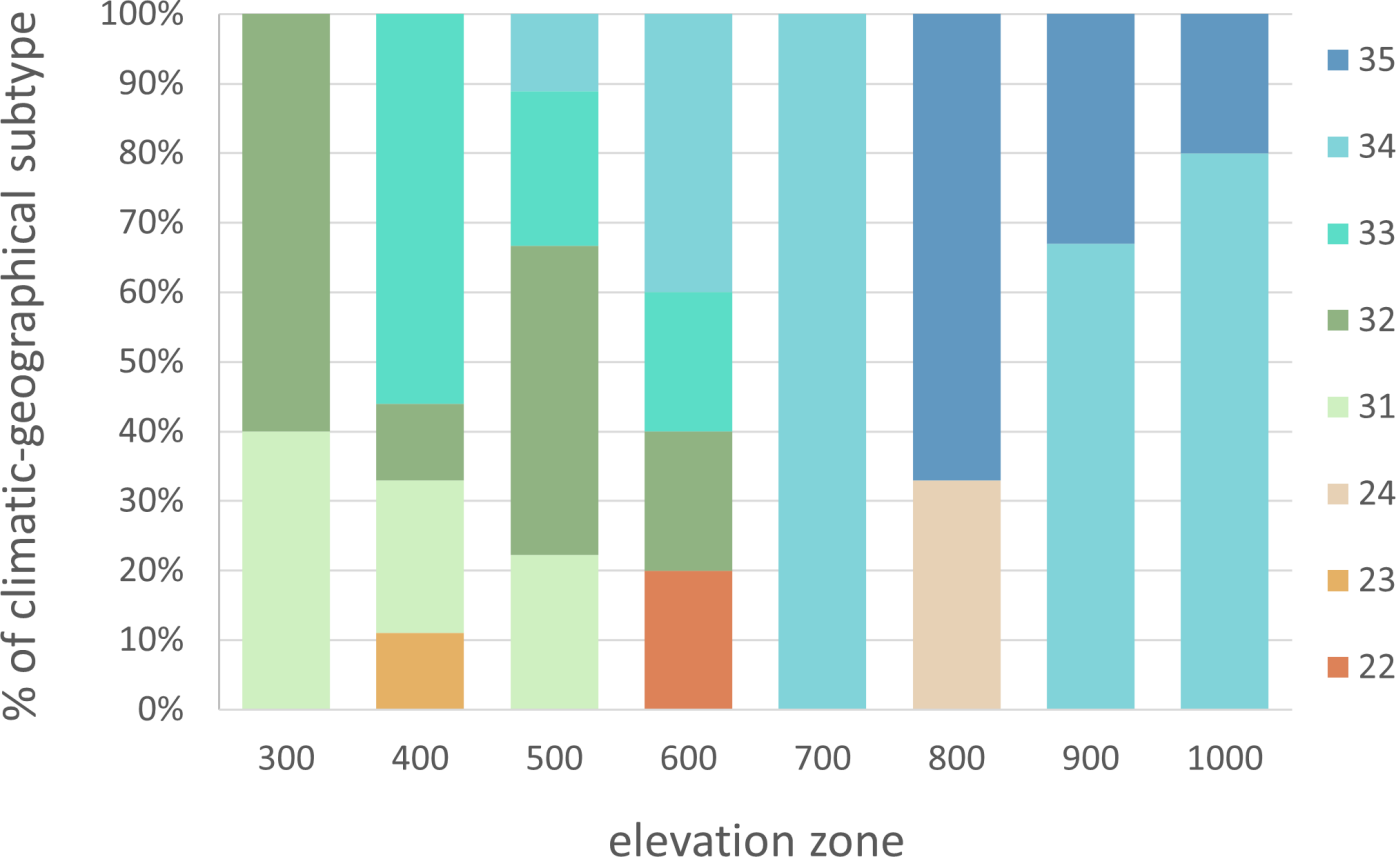
Representation of sites in elevation zones according to climatic-geographical subtypes (22 – Valley, moderately warm, 23 – Valley, moderately cool, 24 – Valley, cool, 31 – Mountain, warm, 32 – Mountain, moderately warm, 33 – Mountain, moderately cool, 34 – Mountain, cool, 35 – Mountain, cold).

The sites were categorized into different types and subtypes based on the climate and geographical classification (**Supplementary Data 1**). The distribution of climatic-geographical subtypes across the elevation zones is depicted in **Figure 2**, indicating the relative percentages. The climate of these areas impacts the phenological response of beech within each elevation zone, which was considered in the subsequent analysis.

**Table 2** provides an overview of the average onset of observed spring (LU - Leaf Unfolding, FL - Full Leaves) and autumn (LC - Leaf Coloring, LF - Leaf Fall) phenological phases, mean earliest and latest onset events​​, and variability over 26 years.

**Table 2:** Statistical characteristics of Day of Year (DOY) for onset of spring and autumn phenological phases from 40 sites, observed in 1996–2021 (LU, Leaf Unfolding; FL, Full Leaves; LC, Leaf Coloring; LF, Leaf Fall; $\bar{x}$ , Mean DOY of onset; ${\bar{x}}_{\min}$ , Earliest interannual mean DOY of onset; ${\bar{x}}_{\max}$ , Latest annual mean DOY of onset; year, year of the earliest/latest interannual mean DOY of onset; *s_x_%*, Coefficient of variation).

Phenological phase	$\bar{x}$	${\bar{x}}_{\min}$ (year)	${\bar{x}}_{\max}$ (year)	*s_x_ %*
LU	115	106 (2014)	125 (1997, 2021)	7.8
FL	120	114 (2014)	131 (2021)	7.4
LC	264	255 (2003)	269 (2020)	4.4
LF	280	275 (2003)	286 (2015, 2020)	5.1

**Figure 3:**
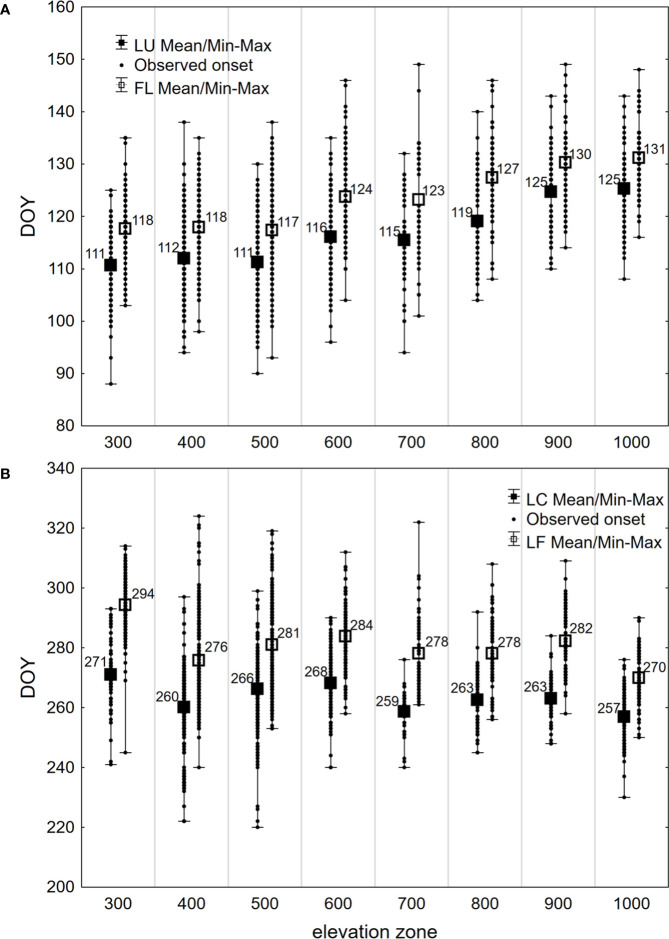
Average, latest (Max), earliest (Min) and all (Observed onset) days of year (DOY) of onset of **(A)** spring phenophases (LU, Leaf unfolding; FL, Full leaves) and **(B)** autumn phenophases (LC, Leaf coloring; LF, Leaf fall) observed in 1996–2021 on 40 sites in the Western Carpathians, grouped by elevation zones.

**Figure 4:**
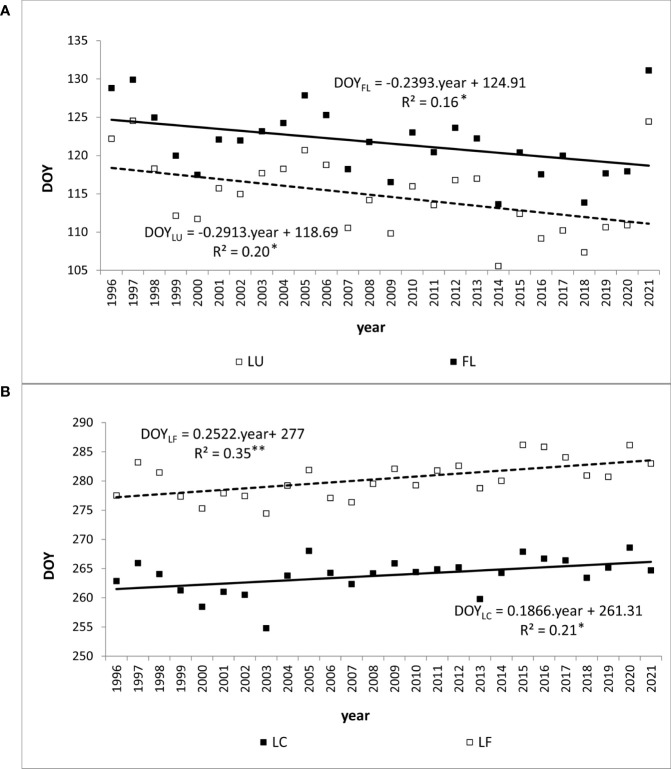
Trend (*p<0.05, **p<0.01) of day of year (DOY) of average onset of **(A)** spring phenophases (LU, Leaf unfolding; FL, Full leaves) and **(B)** autumn phenophases (LC, Leaf coloring; LF, Leaf fall) of European beech observed at 40 sites in Western Carpathians.

During the period under evaluation, the average day of year (DOY) of leaf unfolding (LU) onset for beech across the entire elevation profile was 115 (**Table 2**). The earliest average onset of LU in the entire area was observed on 106 DOY in 2014. The latest average onset was recorded on 125 DOY in 1997 and 2021. As the elevation increased, the onset of leaf unfolding was delayed by approximately -1 to 5 days between neighboring zones (**Figure 3A**). The delay in the onset of the LU phase between the lowest (300) and highest (1000) elevation zones averaged 15 days. A statistically significant (p<0.01) decreasing trend was observed in the development of leaf unfolding over the 26-year monitoring period, indicating an earlier phenophase onset. There was a shift of 7.6 days (equivalent to 2.9 days per 10 years) towards earlier dates within the observed period. That is supported by the negative coefficient of the trend line -0.2913 (**Figure 4A**). The figure shows a significant deviation from the trend of the earlier onset of the phase in 2021. That is caused by an atypical meteorological situation in April. The temperature in this month was under the long-term (1981-2010) average temperature (Bucha et al., 2022). The onset of the LU phase exhibited the highest variability among all the evaluated phenophases (**Table 2**).

The phenological phase of full leaves (FL) typically began on 120 DOY on average. The earliest average onset of FL was observed on 114 DOY in 2014, while the latest average onset occurred on 131 DOY in 2021 (**Table 2**). On average, there was a 14-day difference in the onset of the FL phase between the lowest and highest elevation zones. No significant differences existed between the elevation zones of 300-500 at the onset of the FL phase. However, starting from the elevation zone 600, there was an average delay of 0-6 days as the elevation zone increased (**Figure 3A**). The variability of the onset of FL is similar in both spring phenophases (see Coefficient of Variation in **Table 2**).

The FL phenological phase exhibited a statistically significant (p<0.05) shift towards earlier dates, with an average shift of 6.2 days (equivalent to 2.4 days per 10 years) over the 26 years (**Figure 4A**). This significant shift and the high variability influenced by greater air temperature amplitudes during the spring period make FL a suitable bioindicator of the gradual climate change in the Carpathian region.

**Figure 5:**
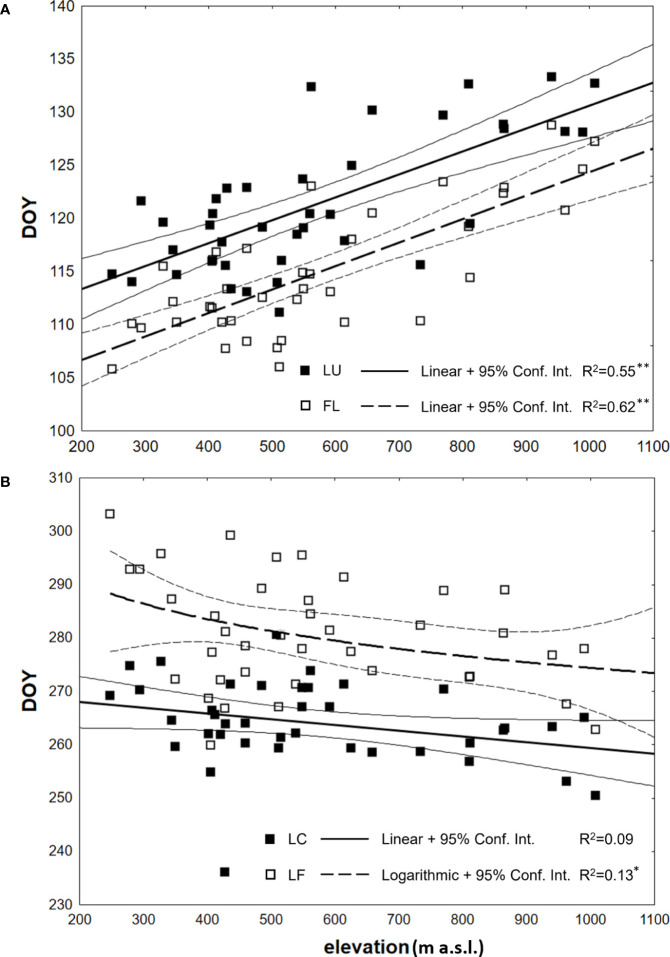
Dependence (*p<0.05, **p<0.01) between the day of year (DOY) of average onset (1996–2021) of **(A)** spring phenophases (LU - Leaf unfolding; FL, Full leaves) and **(B)** autumn phenophases (LC, Leaf coloring; LF, Leaf fall) and elevation of observations, analyzed at 40 sites in the Western Carpathians.

The correlation analysis revealed a statistically significant strong dependence (p<0.01) of the onset of spring phenological phases on elevation, with the coefficient of determination values of 0.62 and 0.55 for LU and FL, respectively (**Table 3**). That indicates that 62% of the change in LU onset and 55% in FL onset can be attributed to variations in elevation. The estimated accuracy of the day of the year (DOY) for LU onset is ± 7.8 days (SE= ± 3.9 days) using the derived regression model with 95% confidence, while for FL onset, the accuracy is ± 9.0 days (SE= ± 4.5 days). A linear model was employed for both spring phenophases (**Figure 5A**), and the b parameter of the regression line yielded a value of 0.0221 or 0.0216. It means that for every 100 m increase in elevation, the onset of spring phenophases is delayed by 2.2 days.

**Table 3:** Regression estimation of the day of the year (DOY) of onset of spring (LU, Leaf Unfolding; FL, Full Leaves) and autumn (LC, Leaf Coloring; LF, Leaf Fall) phenophases based on elevation (a, b, parameters of the model; R^2^, Coefficient of Determination; DF, Degrees of Freedom; SE, Standart Error).

Pheno-phase	Model	*a* parameter	*b* parameter	R^2^	DF	SE [days]
LU	DOY=*a*+*b*.elevation	102.28	0.0221	0.62**	38	± 3.9
FL		109.06	0.0216	0.55**		± 4.5
LC	DOY=*a*+*b*.elevation	270.13	-0.0107	0.09	38	± 7.6
LF	DOY=*a*.ln(elevat.)+*b*	-9.987	343.39	0.13*		± 9.9

**p<0.01; *p<0.05.

### 3.2 Autumn phenology

The autumn vegetative phase of leaf coloring (LC) marks the end of the growing season. In the selected region of the Western Carpathians, the average onset of LC was recorded on 264 (**Table 2**). The earliest average onset of the phenophase occurred on 255 DOY in 2003. The latest average onset in the entire area was recorded on 269 in 2020.

There was a lengthening (p<0.01) of the autumn phenophase by 4.9 days over 26 years (1.9 days per 10 years) (**Figure 4B**). The coloring of leaves started from the highest elevation zones in the entire elevation profile. The average onset differences between neighboring elevation zones ranged from -6 to 10 days (**Figure 3B**). The delay in LC between the lowest and highest elevation zones averaged 14 days. The phenological elevation gradient of the phase is estimated at a 1.1-day delay per 100 m, as indicated by the *b* parameter (**Table 3**). A consistent and gradual trend of a later phenophase onset with decreasing elevation was not observed in all neighboring elevation zones. This variation can be attributed to different climatic-geographical types of phenological stations in each elevation zone. The early average onset of the phase in elevation zone 400 is influenced by a higher representation of the mountain type, moderately cool climatic-geographical subtype (code 33), which is significantly more prevalent than in the neighboring zones 300 and 500 (**Figure 2**). We also observe a similar deviation between zones 500 and 600 and 800 and 900. However, there is no significant difference between the representation of climatic-geographical types between the mentioned, so they result from other drivers’ actions.

Leaf fall (LF) of European beech in the study area typically began on 280 DOY, on average (**Table 2**). The earliest average onset was observed on 275 DOY in 2003. The latest average onset occurred on 286 DOY in 2015 and 2020. Over the 26 years of phenophase records, a statistically significant (p<0.01) delay of 6.6 days was observed, indicating a delay in the onset of the LF phase by 2.5 days per 10 years (**Figure 4B**). The entire elevation profile exhibited a 25-day difference in average onset between the lowest and highest elevation zones (**Figure 3B**). The differences in onset between neighboring zones ranged from -6 to 19 days. The variability of LF onset in elevation zones during the 26-year observation period was similar to that of the previous autumn phase (see Coefficient of Variation in **Table 2**) and lower than observed in the spring phenophases.

The onset of the leaf coloring (LC) phase did not exhibit a statistically significant correlation with elevation (**Table 3**). However, in the leaf fall (LF) phase, the correlation coefficient with p<0.05 differed significantly from zero. Compared to the spring phenophases, the relationship’s strength was notably lower based on the coefficients of determination. The influence of elevation on the LF phase during autumn accounted for only 13% of the variation in the day-of-year (DOY) values for the onset of the phenophase. The accuracy of estimating the DOY of these phenophases from the regression models mentioned earlier was lower. For the LF phenophase, a more suitable regression model was found using a logarithmic function with negative values of the parameter *a* (**Table 3**). Its course is depicted in **Figure 5B**. The differences in the earlier onset of LF with increasing elevation were more pronounced at lower elevations and decreased at higher elevations. The investigated phenological elevation gradient indicated an earlier onset by 2.9 days per 100 m between 300 and 400 m a.s.l., 1.5 days per 100 m between 600 and 700 m a.s.l., and 1.1 days per 100 m between 900 and 1000 m a.s.l.

### 3.3 Trends in growing season duration

**Figure 6:**
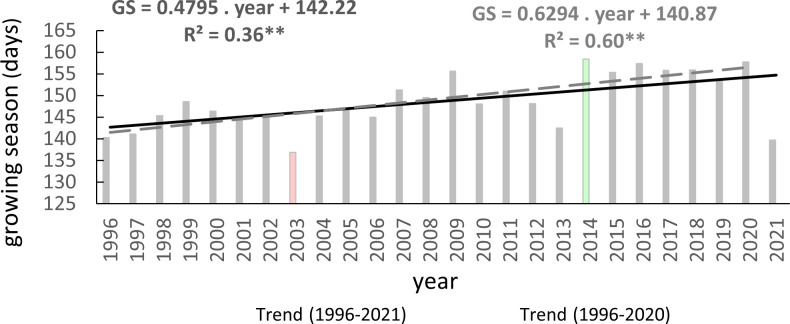
Average length of growing season (GS) of European beech observed on 40 sites in the Western Carpathians (2003 – year of the shortest GS, 2014 – year of the longest GS) and its trends (**p<0.01) for 1996–2021 and 1996–2020.

One reliable method for assessing climate change’s impact on beech phenology is through long-term monitoring of the growing season (GS) duration. In the assessed area, the average length of the growing season was 149 days. The shortest average growing season was recorded in 2003 (137 days), the longest occurred in 2014 (158 days), and was followed by another six-long GS (**Figure 6**). These years were classified as extremes in terms of air temperature and precipitation during the summer, with 2003 being exceptionally dry and warm compared to 2014, which experienced more typical climatic conditions (Lapin et al., 2016; Škvareninová et al., 2018). The lengthening trend of the growing season was statistically significant at p<0.01, with a correlation coefficient of 0.6. Over the entire monitoring period, the growing season was extended by an average of 12 days, equivalent to 4.8 days per 10 years.

The average duration of the growing season varied by 28 days between the lowest (300) and highest (1000) elevation zones (**Table 4**). Generally, the average duration of the growing season increased with decreasing elevation. However, there was an exception in elevation zone 400, where the average growing season was shorter than neighboring zones. Zone 400 and zone 600 exhibited high climatic-geographical heterogeneity of the sites (**Figure 2**), leading to this bias. The observation further demonstrates the heterogeneity of phenological stations in zone 400, where the absolute longest growing season in 1999 (195 days) and the shortest one in 2021 (100 days) were recorded in this elevation zone.

**Table 4:** Average length, the extremes in the growing season, and the lengthening trend in elevation zones ( $\bar{x}$ - Mean, *x_min_* - the Shortest Duration, *x_max_ -* the Longest Duration, R - Coefficient of Correlation).

Elevation zone	$\bar{x}$ (days)	*x_min_* (days)	*x_max_* (days)	trend of gain (days per 26 years)	R
300	160	144	170	17.6	0.70**
400	148	135	159	7.5	0.36
500	155	139	165	9.9	0.47*
600	152	133	172	8.8	0.33
700	144	132	162	17.2	0.64**
800	143	128	160	18.3	0.59**
900	138	120	151	18.7	0.71**
1000	132	124	144	15.5	0.70**

**p<0.01; *p<0.05.

A statistically highly significant trend (p<0.01) of lengthening the growing season was observed in five elevation zones, and it was significant (p<0.05) in one zone (**Table 4**). However, a statistically insignificant trend of lengthening the growing season was found in elevation zones 400 and 600. The most pronounced lengthening trend was observed in elevation zones 900 and 300 (**Table 4**). These results indicate a lengthening of the growing season in most elevation zones of the beech occurrence in the Western Carpathians as a response to warming, except for elevation zones 400 and 600.

**Figure 7:**
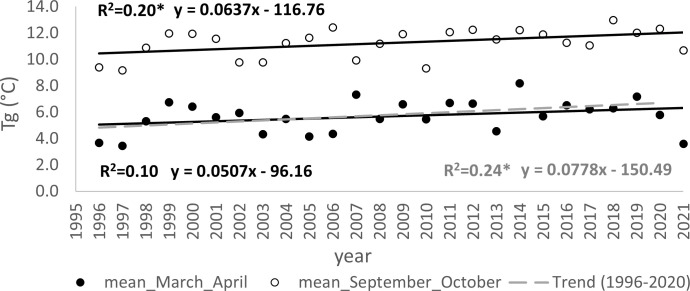
Trends (*p<0.05) in development of mean of two-month average temperature before and during the onset of spring phenophases (March, April) and autumn phenophases (September, October) for the periods 1996-2021 and 1996–2020; the mean determined from measurements at four meteorological stations in the elevation gradient of the Western Carpathians, ranging from 231–906 m a s.l.

The warming trends during the critical months impacting the initiation (March and April) and conclusion of the growing season (September and October) are illustrated in **Figure 7**. In the period spanning 1996-2021, a statistically significant increase in 2-monthly average temperatures during autumn was confirmed (p<0.05), with a rate of 0.06°C per year. Conversely, for the spring months, this trend was either inconclusive or only verified after the exclusion of the extreme value noted in the 2-month average of 2021. Within the timeframe of 1996-2020, the statistical test affirmed the significance (p<0.05) of rising temperatures during the spring months, showing an increase of 0.08°C per year.

**Figure 8:**
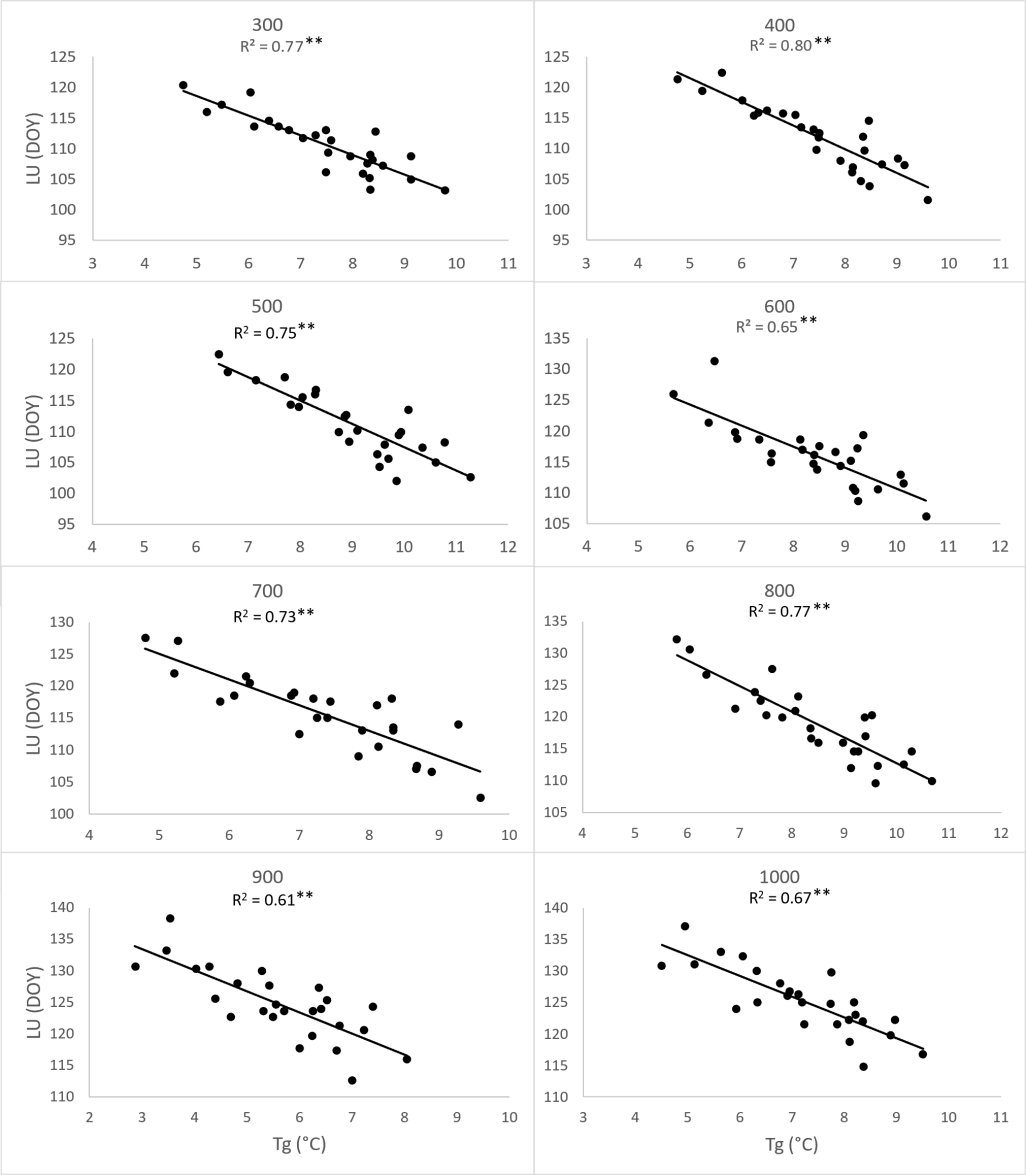
Dependence (**p<0.01) of day of year (DOY) of leaf unfolding (LU) average onset (1996-2021) on the 26-years mean (Tg) of two-month (March, April) average temperatures, derived for elevation zones 300-1000 at 40 sites in the Western Carpathians.

**Figure 9:**
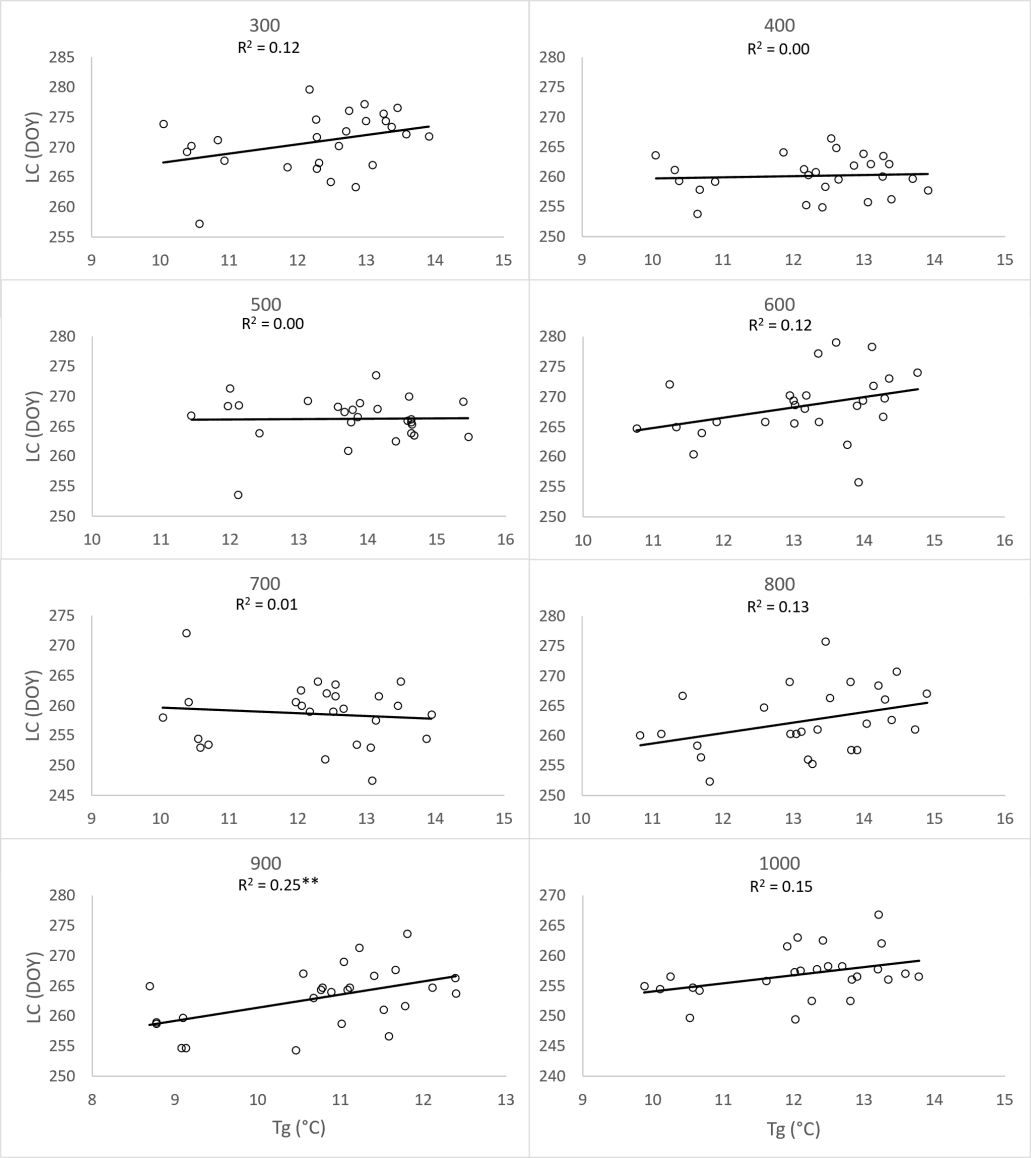
Dependence (**p<0.01) of day of year (DOY) of leaf coloring (LC) average onset (1996-2021) on the 26-years mean (Tg) of two-month (September, October) average temperatures, derived for elevation zones 300-1000 at 40 sites in the Western Carpathians.

To substantiate the impact of increasing temperatures on the prolongation of the growing season, we assessed the correlation between the day of year (DOY) for the onset of Leaf unfolding (LU) (**Figure 8**) and Leaf coloring (LC) (**Figure 9**) with the 2-month average temperatures of March and April, as well as September and October. The analysis was conducted separately for each elevation zone. Concerning the commencement of the growing season, the influence of average temperatures in March and April emerged as a significant driver (p<0.01) for the earlier onset of this spring phenophase across all zones from 300-1000. However, increasing temperatures in September and October did not exhibit an impact on the conclusion of the growing season. Except for the altitude zone 900, other zones did not demonstrate a significant influence of temperature on the shift of the growing season’s end (**Figure 9**).

**Figure 10:**
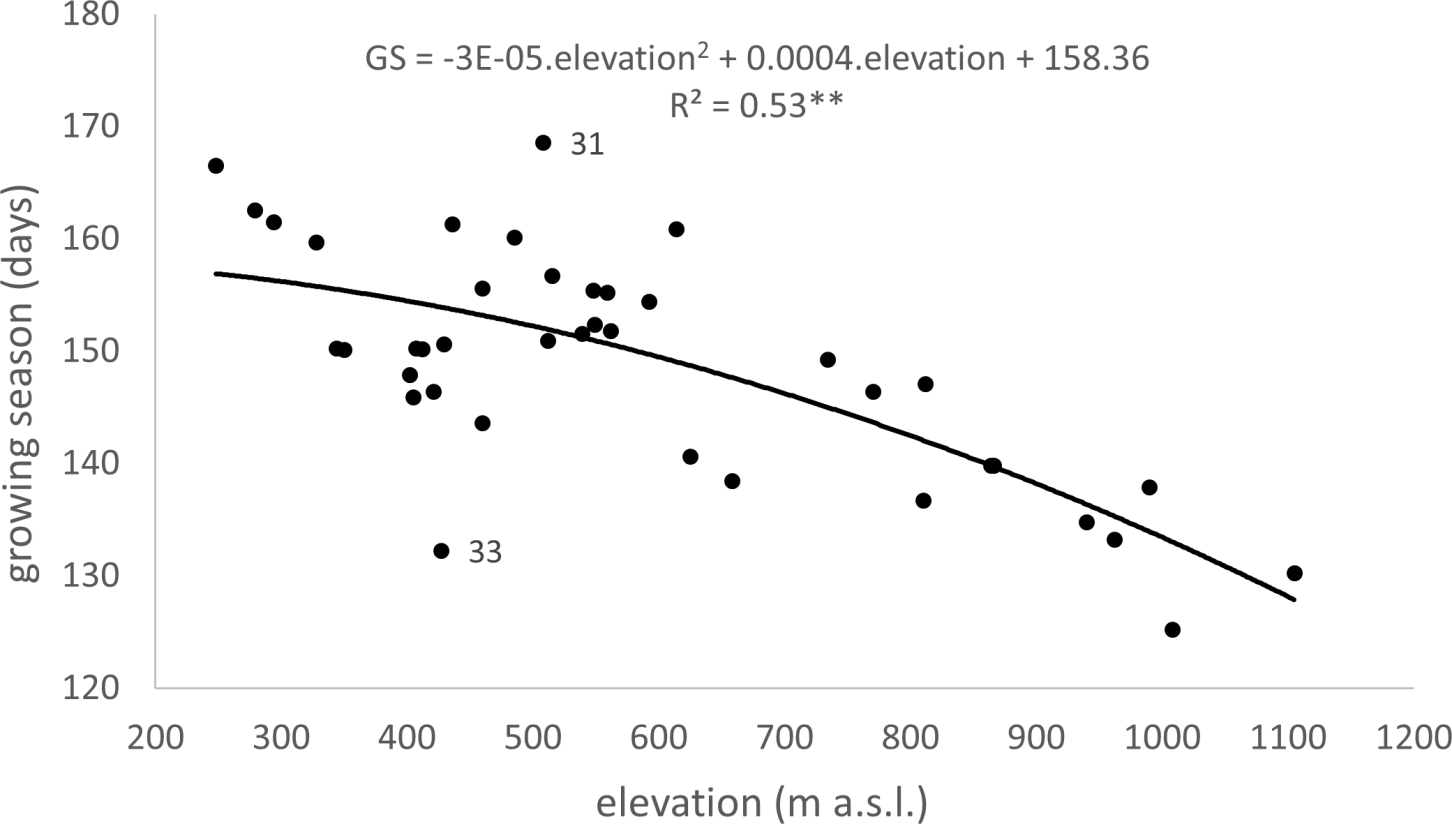
Dependence (**p<0.01) of long-term (1996–2021) mean of growing season (GS) duration on the elevation at 40 sites in the Western Carpathians (R^2^ – Coefficient of Determination, 31 – Mountain climatic-geographical type and warm subtype, 33 – Mountain climatic-geographical type and moderately cool subtype).

**Figure 11:**
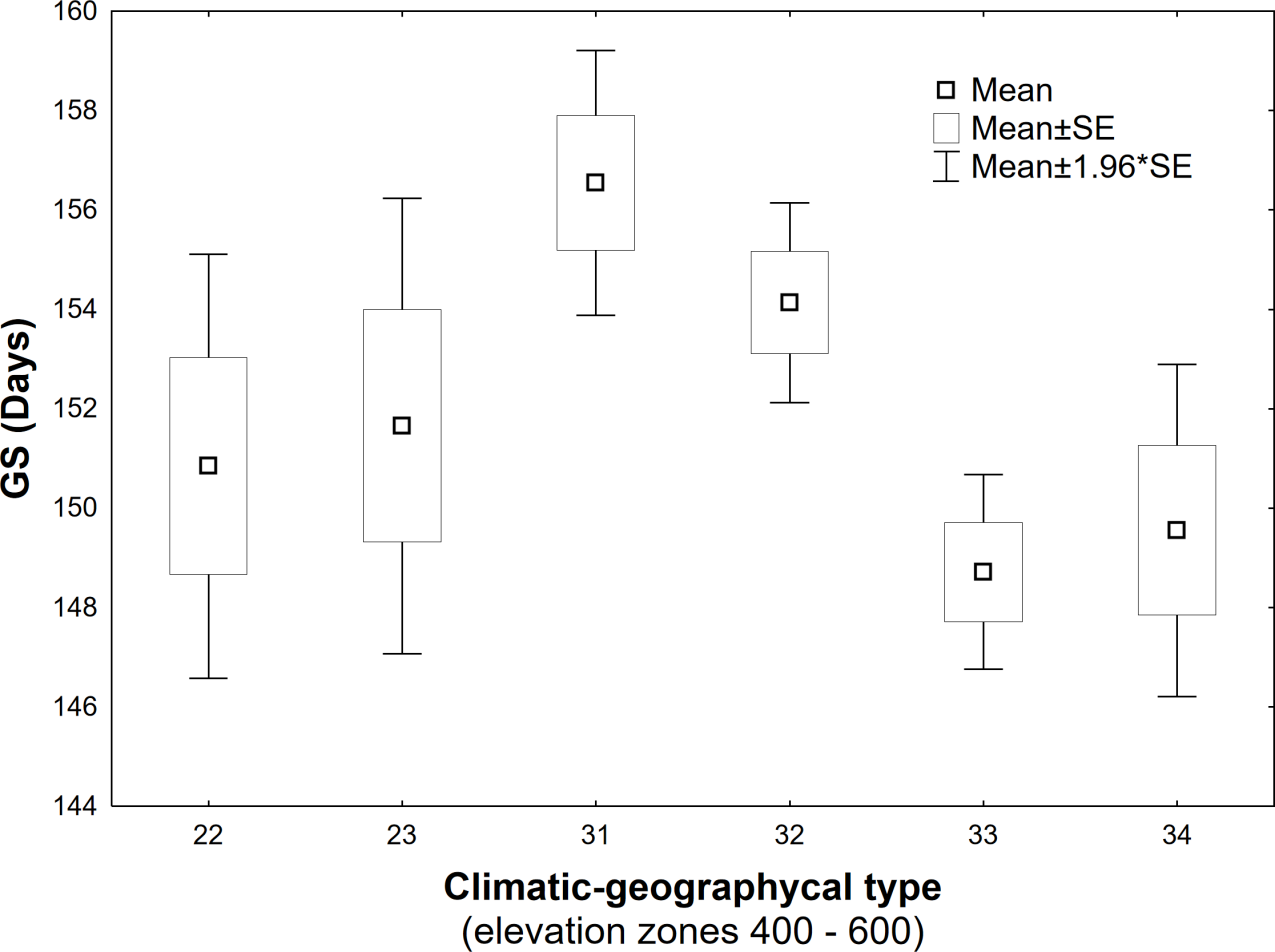
Differences in long-term (1996-2021) mean of the growing season (GS) duration between climatic-geographical subtypes occurring within elevation zones 400-600 (22 – Valley, moderately warm, 23 – Valley, moderately cool, 31 – Mountain, warm, 32 – Mountain, moderately warm, 33 – Mountain, moderately cool, 34 – Mountain, cool).

The elevation gradient of the growing season duration is illustrated in **Figure 10**. According to the quadratic function, elevation accounts for 53% of the variability in GS duration (R^2 =^ 0.53). The decrease in GS duration with increasing elevation is more gradual in the lower elevations of 300–600 m, with a gradient of 1.7–2.7 days per 100 m. At 700–1000 m elevations, the gradient increases to 3.3–4.8 days per 100 m. There is a more significant variation in the mean duration of the growing season in the elevation range of 400-600 m (**Figure 10**), corresponding to a more significant variation in climatic and geographical conditions at these sites (**Figure 2**, elevation zones 400, 500, 600). The marked variation in growing season duration (GS) values, indicated by the climatic-geographical code 31 (mountain type, warm subtype) and 33 (mountain type, moderately cool), represents sites with slight differences in elevation (81 m). However, the average duration of the growing season over the 26 years significantly differs between these sites ( $\bar{GS_{33}}$ =132 days, $\bar{GS_{31}}$ =169 days). ANOVA and *post-hoc* test confirmed a significant (p<0.05) difference in average growing season (GS) duration within some climatic-geographical types evaluated for 23 sites located within elevation zones from 400 to 600. The average GS duration in the mountain, moderately cool subtype (code 33) and mountain, cool (code 34) is shorter by 5.4 days and 4,6 days, respectively, compared to the mountain, moderately warm subtype (code 32) and 7.8 days and 7.0 days respectively compared to the mountain, warm subtype (code 31) (**Figure 11**). For other represented subtypes, the valley, moderately warm (code 22) and valley, moderately cool (code 23), were not confirmed significant differences in GS duration due to their big variance within the subtype.

Based on the findings, we have discovered that climatic-geographic types represent another factor influencing the duration of growing season, taking into account the conditions of relief anomalies. This factor explains the disruption of the elevation gradient sequence between certain elevation zones. This leads to a situation where the growing season lasts longer in elevation zone 500 than in lower one 400 (**Table 4**). We attribute this to the fact that in the elevation zone 400 the sites with phenological observations were represented up to 55% by subtype 33 mountain, moderately cool), and in elevation zone 500 the warmer subtypes 31 and 32 (mountain, warm and moderately warm) dominated with a representation of 66% (**Figure 2**).

The results indicate that phenological phases serve as important bioindicators of climate change, even when a sufficient amount of meteorological data is not available. Shifts in phenophase trends result in an extension of the growing season, which serves as evidence of climate change.

## 4 Discussion

Indicators for objectively assessing climate change in European beech habitats include trends in the onset of phenological phases and the length of the growing season.

Our findings for the spring phenological phases LU and FL, covering 1996-2021, reveal a trend of their earlier onset. These results are consistent with the work of Schieber (2006) in the central part of the Inner Western Carpathians, which also showed a shift towards an earlier onset of spring phenological phases. Similar trends of earlier onset have been observed in several European tree species, as Bigler and Vitasse (2019) documented in the European phyto-phenological database. Spring phenology of temperate trees has advanced worldwide in response to global warming. According to Wenden et al. (2020), the onset of spring phases is a response to higher spring temperatures, which is more pronounced in colder regions as an evident manifestation of ongoing climate warming.

Earlier onset of spring phenophases was observed in Western European regions before 2000. Our results suggest that changes are also occurring at present. Ahas et al. (2002) found a shift of 3-4 days per 10 years during the period 1951-1998, while Jablonska et al. (2015) confirmed an earlier onset of spring phases of nine tree species by 1.8-3 days per 10 years in Poland between 1951 and 1990. A study conducted in mountainous areas above 1000 m a.s.l. in Slovenia recorded an increasing trend of earlier onset by 1.52 days per 10 years, while no changes were observed in lower elevations (Čufar et al., 2012). Dewan et al. (2020) highlight the spatiotemporal fluctuations of spring air temperature due to ongoing climate change, resulting in a rapid phenological response in beech. We observed such a reaction in 2014. Our results correspond to the early onset of LU in the north-eastern part of Austria in the same year (Dolschak et al., 2019). This phenological extreme makes it possible, even without temperature data, to confirm the agreement of temperature conditions with other mountain regions of Europe. It is a manifestation of the dynamics of temperature changes as one of the accompanying phenomena of climate change.

We focused on mountain areas within this region Western Carpathians to ensure comparability for our study. Older research by Ermich (1960) indicated that leaf unfolding at an elevation of 1000 m a.s.l. occurred at the turn of the third and fourth week of May. Our results at this elevation demonstrate an average onset in the first week of May. These phenological differences highlight the changing temperature conditions, even at higher elevations.

The autumn phenophases LC and LF exhibited a trend of later onset and lower variability compared to the spring phases. A statistically significant delay of 6.6 and 4.9 days was observed for the autumn phases over 26 years (equivalent to 2.5 and 1.9 days per 10 years, respectively). Schieber et al. (2017) recorded a delay of the LC phase by 4.3 days per 10 years in the submontane European beech forest of the Inner Western Carpathians from 1995 to 2015. Our results demonstrate that the onset of autumn phenophases varied across individual elevation zones. Notably, a substantial early onset of LC was observed at the 400 m elevation zone, likely influenced by the moderately cold mountainous climate and the effect of cold air from the inverse valleys. Similar phenological responses in mountainous locations have also been reported by Schuster et al. (2014).

The dynamics and increased occurrence of extreme weather events are accompanying signs of climate change (Ziernicka-Wojtaszek, 2021). Lapin et al. (2016) reported more frequent extreme summer temperatures and heatwaves in the monitored area during 1995-2015 (specifically in 1995, 2003, 2013, and 2015). In specific years (2003, 2013), these extreme events led to an earlier onset of LC in our study area, particularly in lower locations. Early leaf coloring in beech trees was also observed in other Central European countries in 2003 (Seletkovič et al., 2009). Extreme weather events can impact the earlier onset of LC and consequently shorten the beech growing season in the study area. Conversely, in years with favorable weather (such as in 2014), the autumn phases occurred later, as confirmed by Lukasová et al. (2020). That aligns with our results showing the most extended growing season in 2014 for the entire monitoring period.

Climate change also affects the phenological elevation gradient. We found phenological elevation gradients of 2.2 days per 100 m for the spring phenological phases during 1996-2021. This gradient is slightly lower than the gradient reported by the beech phenological network in Slovenia (Čufar et al., 2012), which documented a shift in the gradient of 2.6 days. Schieber et al. (2013) reported higher gradients (2.8-3 days per 100 m) for the spring phenophases of beech in the Inner Western Carpathians, which can be attributed to a wider elevation range of 200-1400 m and a shorter period. A comparison of these results suggests a reduction in the elevation gradient for spring phenophases with ongoing climate change.

The elevation gradient of leaf coloring (LC) varied along the elevation profile, as noted by Bialobok et al. (1990). Up to 500 m elevation, a phase shift of 2 days per 100 m towards earlier onset. Above 500 m, the gradient increased to 3-4 days per 100 m elevation. Our results demonstrated a less pronounced and linear development of the LC gradient, with a shift of 1.1 days per 100 m across the entire elevation profile. Popescu and Sofletea (2020) also found a more significant elevation gradient of 1.8-2.4 days per 100 m in the Western Romanian Carpathians at 200-1200 m elevations. They observed a non-linear gradient development, similar to Bialobok et al. (1990), but with greater shortening at higher elevations. These differences in results may be influenced by the presence of a sub-Mediterranean climate in lower areas up to 500 m, which was not present in our study area. For the elevation interval of 200-1400 m in the central part of the Carpathians during the years 2007-2011, Schieber et al. (2013) reported a consistent gradient of 1-1.8 days per 100 m, which aligns with our findings. During the autumn period was lower variability in the onset of phenophases. This finding is also supported by the work of Čufar et al. (2012).

Our research investigated a non-linear course of the elevation gradient (1.1-2.9 days per 100 m) for the onset of the leaf fall (LF) phase. Schieber et al. (2013) reported a 1-1.78 days per 100 m gradient, consistent with our results for elevations above 500 m. However, we observed a higher gradient for the lower elevation of beech occurrence. The more significant difference in the LF gradient up to 500 m elevations could be influenced by the development caused by further environmental over the last decade, which was not considered in the evaluation by Schieber et al. (2013). Further research is needed to confirm these findings.

Analysis of tree data from the International Phenological Gardens network for 1969-1998 revealed that the average length of the growing season in similar geographical conditions ranged from 132 to 146 days, depending on the tree species. The start of the growing season shifted earlier by an average of eight days (2.7 days per 10 years) (Chmielewski and Rötzer, 2001, 2002). These trends toward a longer growing season are attributed to global warming (Ahas, 1999; Beaubien and Freeland, 2000). Our results indicate a more significant impact of warming in the past two decades. Similar findings of a growing season duration of 154 days have been reported for these areas by Schieber et al. (2013). Scientific projections suggest that further warming could extend the beech growing season by 20 days (3.3 days per 10 years) in the next 60 years (Prislan et al., 2019). These trends of earlier spring and later autumn phenophases onset are likely to influence the competitive balance among species (Vitasse et al., 2011). Beech populations at lower elevations may experience a shorter growing season due to drought conditions (Vitasse et al., 2010). Vitase and Basler (2013) assert that beech phenology follows a non-linear trend across biogeographical gradients, such as changes in elevation and photoperiod. We did not deal with the influence of photoperiodism in our work, as it is an area with minor differences in longitude (48°13’-49°31’N) of individual sites, where this influence is insignificant. Our analyses confirmed that the duration of the growing season shortens with increasing elevation and present similar results reported by Popescu and Sofletea (2020) regarding the shortening of the growing season by 4 days per 100 m in the region of the Western Romanian Carpathians. Extreme changes of weather can cause sudden changes in the length of the growing season (Schueler and Liesebach, 2015). For instance, in 2003 our phenological results indicate a shortening of the growing season by up to 12 days compared to the similar results (14 days) (Massonnet et al., 2021).

Monitoring climate change using phenological elevation gradients will provide valuable insights into the development and response of forest trees to climate change and trends in elevation and spatial distribution.

## 5 Conclusion

Phenological observations of tree species serve as a suitable bioindicator for objectively assessing climate development, utilizing the trends in the onset of phenological phases, phenological elevation gradients, and the length of the growing season. This study documented the changes in selected spring and autumn vegetative phases of European beech (*Fagus sylvatica* L.) based on long-term observations (1996-2021) across eight elevation zones characterized by diverse geographical and climatic conditions. The heterogeneity of climatic-geographical types between elevation zones helped to explain the atypical onsets of phenological phases concerning elevation.

The findings confirm a statistically significant trend of earlier onset for spring phenophases (LU, FL). The phases exhibit high variability, reflecting changes and dynamics in air temperature over the entire period and within individual years. In contrast, the trend of later onset for autumn phases proved less significant. The dependence of the onset of leaf coloring on the 2-month mean of temperatures before and during the months of the onset of this phenophase (September and October) was also not proven, and in general, the onsets of autumn phenophases are less variable.

The consequence of changes in the onset of spring and autumn phenophases is an elongation of the growing season throughout the entire elevation profile of European beech occurrence in the Western Carpathians. On average, the growing season of European beech in the study period lasted 149 days but shortened with increasing elevation. The intensity of shortening becomes more pronounced as elevation increases. Within the elevation range of 400-600 m, the significant heterogeneity of climatic and geographical conditions resulted in substantial variability in the duration of the growing season among locations with slight differences in elevation. The long-term trend indicates an average lengthening of 4.8 days per 10 years over the monitoring period. Our findings confirm that the lengthening of the growing season due to warming, as an expression of climate change, is predominantly attributed to the warming in the spring months.

By extending the growing season, it is assumed that the beech area will be changed to locations with optimal environmental conditions, especially in terms of adverse climatic events (late spring frosts, drought) during the growing season. However, years with climatic extremes, such as 2003, 2013, and 2021, led to significant growing season shortening. That has a significant impact on the future of beech forest management.

The phenological behavior of European beech provides valuable indicators of its response to current and long-term local climate changes. This study’s contribution lies in utilizing a climatic-geographical classification to evaluate phenological behavior, accounting for the influence of elevation and the specific climatic conditions resulting from the terrain relief. Doing so provides insights into the specific biological conditions of the European beech environment in the Western Carpathians.

The impact of climatic-geographical anomalies on the phenological processes of European beech may lead to changes in its distribution in the climatic-extreme areas within the Western Carpathians. The onset of phenophases influenced by specific climate conditions could find future applications in forestry when selecting sites where beech is less susceptible to late spring frosts. Valuable data obtained through ground-based observation methods can be utilized in modeling the further phenological development of beech under climate change conditions. Phenological data will also be useful in retrospectively assessing weather extremes in individual years by comparing phenological phases when meteorological data are unavailable.

## Data availability statement

The raw data supporting the conclusions of this article will be made available by the authors, without undue reservation.

## Author contributions

JnS, RS, and JrS contributed to the conception and design of the study. JnS, ZS organized the database. RS performed the statistical analysis. JnS and RS wrote the first draft of the manuscript. JnS, RS, JV, and JrS wrote sections of the manuscript. RS and JV contributed to the manuscript revision, and read, and approved the submitted version. All authors contributed to the article and approved the submitted version.
