# Supplementary material for: Phenological response of European beech (Fagus sylvatica L.) to climate change in the Western Carpathian climatic-geographical zones
Source: Front Plant Sci. 2024 Apr 3;15:1242695. doi: 10.3389/fpls.2024.1242695 (PMC11022973; doi:10.3389/fpls.2024.1242695)
Supplement: Supplementary file 1 [file DataSheet_1.docx]

Supplementary Material

Phenological Response of European Beech (Fagus sylvatica L.) to Climate Change in the Western Carpathian Climatic-Geographical Zones

Jana Škvareninová, Roman Sitko, Jaroslav Vido, Zora Snopková, Jaroslav Škvarenina

*** Correspondence:** Roman Sitko: sitko@tuzvo.sk

# Supplementary Data

Supplementary Material – Characteristic of beech phenological stations (**Supplementary Data 1.xlsx** file)

# Supplementary Figures and Tables

## Supplementary Figures


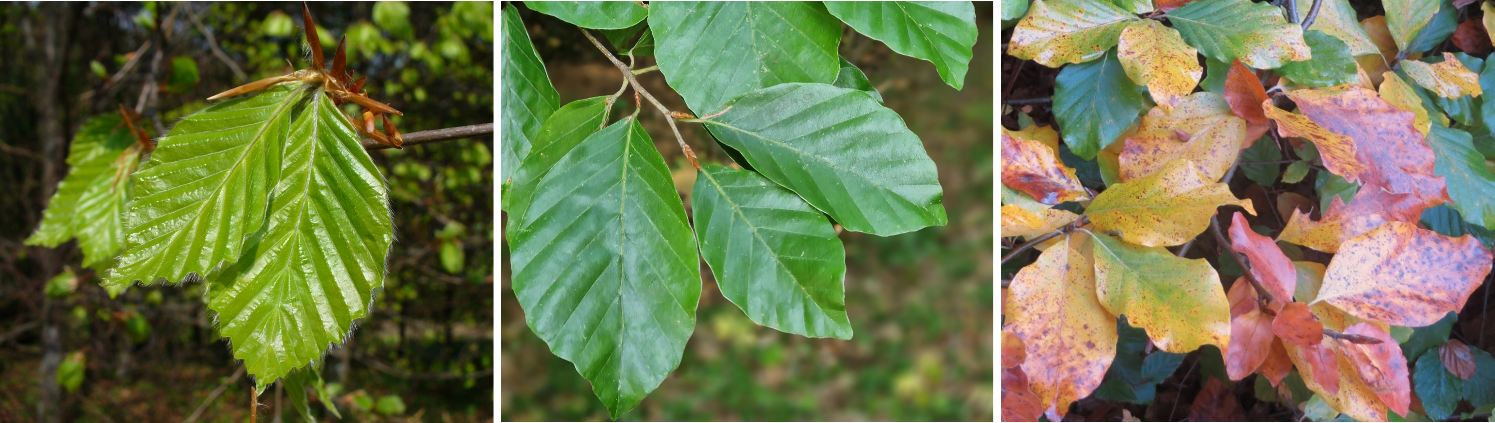


a) b) c)

**Supplementary Figure 1.** Images of observed phenological phases; 1a) Leaf unfolding, 1b) Full leaves, 1c) Leaf coloring
